# ZnO/Graphene Oxide on Halloysite Nanotubes as a Superabsorbent Nanocomposite Photocatalyst for the Degradation of Organic Dyes

**DOI:** 10.3390/nano13131895

**Published:** 2023-06-21

**Authors:** Jongik Park, Hyungwook Lee, Keonku Lee, Sieun Noh, Soyeong Jin, Jungho Jae, Youngdo Jeong, Jaegeun Noh

**Affiliations:** 1 Department of Convergence of Nanoscience, Hanyang University, 222 Wangsimni-ro, Seongdong-gu, Seoul 04763, Republic of Korea; siy171@naver.com; 2 Department of Chemistry, Hanyang University, 222 Wangsimni-ro, Seongdong-gu, Seoul 04763, Republic of Korea; winning_07@naver.com (H.L.); leekku10@gmail.com (K.L.); sieun.noh@kakao.com (S.N.); truejin@hanyang.ac.kr (S.J.); 3 Center for Biomaterials, Biomedical Research Institute, Korea Institute of Science and Technology (KIST), Seoul 02792, Republic of Korea; 4 School of Chemical and Biomolecular Engineering, Pusan National University, Busan 46241, Republic of Korea; jh.jae@pusan.ac.kr; 5 Department of HY-KIST Bio-Convergence, Hanyang University, 222 Wangsimni-ro, Seongdong-gu, Seoul 04763, Republic of Korea; 6 Research Institute for Convergence of Basic Science, Hanyang University, 222 Wangsimni-ro, Seongdong-gu, Seoul 04763, Republic of Korea

**Keywords:** photocatalyst, halloysite nanotubes, graphene oxide, zinc oxide, nanocomposite, photocatalytic effect, degradation, organic dyes

## Abstract

Using renewable photocatalysts for pollutant degradation represents a promising approach to addressing environmental water challenges by harnessing solar energy without additional energy consumption. However, for the practical use of photocatalysts, it is necessary to improve catalyst efficiency, considering cost and biocompatibility. In this study, we developed a new superabsorbent photocatalyst for the degradation of organic dyes in water. Our photocatalyst comprises halloysite nanotubes (HNTs) with a large outer diameter and Si-O and Al-O groups on the outer and inner surfaces, respectively; graphene oxide (GO) possessing numerous sp2 bonds and light-conductive properties; and ZnO, which can degrade organic molecules via a photon source. By exploiting the superabsorbent properties of GOs for organic dyes and stabilizing ZnO nanoparticles on HNTs to inhibit aggregation, our photocatalysts demonstrated significantly improved degradability compared to ZnO nanoparticles alone and combinations of ZnO with HNTs or GO. The structural characteristics of the nanocomposites were characterized using SEM, EDX, Raman spectroscopy, and XRD. Their enhanced photocatalytic activity was demonstrated by the degradation of rhodamine b in water, showing 95% photodegradation under UV illumination for 60 min, while the ZnO nanoparticles showed only 56% dye degradation under the same condition. Additionally, the degradation rate was enhanced by four times. Furthermore, the catalysts maintained their initial activity with no significant loss after four uses, showing their potential for practical implementation in the mass purification of wastewater.

## 1. Introduction

Water contamination caused by organic dyes used in textile industries represents a significant environmental concern. In order to address this issue, various methods have been explored for removing organic dyes from industrial wastewater. Among these methods, the use of photocatalysts to degrade organic dyes has garnered significant attention, primarily due to the advantages offered by solar energy utilization [1,2,3,4,5,6]. Zinc oxide (ZnO), in particular, has emerged as a promising semiconductor material for photocatalytic applications. It possesses a wide band gap of 3.17 eV and a large exciton binding energy of 60 meV. These properties make ZnO well suited to the photocatalytic degradation of organic dyes [6,7,8,9,10,11]. Importantly, ZnO exhibits photocatalytic activity without suffering from photocorrosion, a phenomenon that can hinder the long-term stability and performance of other materials used for photocatalysis. By harnessing the energy from sunlight, ZnO-based photocatalysts can efficiently initiate the degradation process of organic dyes, thereby mitigating the environmental impact caused by wastewater from textile industries. The utilization of ZnO’s inherent photocatalytic properties provides a sustainable and environmentally friendly approach for treating and purifying wastewater, facilitating the removal of harmful organic dyes and reducing their detrimental effects on aquatic ecosystems. In particular, one-dimensional ZnO nanostructures with excellent crystallinity and no defects are of particular interest within the realm of ZnO nanomaterials. This is because they possess distinctive and inherent intrinsic characteristics in terms of their chemical, electrical, physical, and mechanical properties, which distinguish them from bulk and thin-film ZnO materials.

In order to achieve efficient degradation of organic dyes, it is necessary to address the tendency of ZnO nanoparticles to aggregate in water, despite their relatively larger surface areas compared to bulk materials [12,13,14]. To counteract this aggregation issue, the use of a supporting material becomes crucial. As a supporting material, halloysite (an aluminosilicate clay with a kaolin group) nanotubes (HNTs) are broadly used in various applications such as catalysis, adsorption, and functional sensing materials, due to their nanosized tubular structures [15,16,17,18,19,20,21,22,23,24,25]. The outer surface of HNTs is characterized by a negatively charged silicate layer, while the inner surface is composed of a positively charged alumina layer [26,27,28]. This distinctive property allows for effective adsorption of ZnO onto the surface of HNTs, thereby preventing the aggregation of ZnO. By immobilizing ZnO onto HNTs, the resulting composite achieves stabilization in water, facilitating its practical application as a photocatalyst. While achieving stability in water is an important step, enhanced photocatalytic activity is also imperative for practical applications [29,30,31,32,33,34,35,36,37]. The combination of ZnO and HNTs not only provides the necessary stability but also results in improved photocatalytic performance. This enhanced activity arises from the synergistic effects between ZnO and HNTs, where the unique properties of both materials work in tandem to enhance the degradation efficiency of organic dyes. By utilizing the nanosized tubular structure of HNTs as a support for ZnO NPs, the composite material not only prevents NP aggregation but also promotes effective utilization of solar energy for the degradation of organic dyes. This advancement in photocatalytic activity holds great promise for practical applications, facilitating the development of efficient and sustainable methods for the treatment and purification of wastewater contaminated with organic dyes. Additionally, although GO as a nanomaterial has shown great adsorbent properties in relation to organic dyes, it has not been applied to ZnO on HNT nanocomposites. Exclusively, the diverse roles of GO in heterogeneous photocatalysis arise from its unique structure and surface chemistry characteristics [38]. GO serves not only as a precursor for graphene synthesis but also as a “macromolecular surfactant”, facilitating the dispersion of insoluble materials [39]. The two-dimensional structure of GO allows it to function as a growth template, influencing the synthesis of composite materials with distinct morphologies [40]. Additionally, GO can serve as a building block for the preparation of three-dimensional graphene-aerogel-supported photocatalysts [41]. Moreover, appropriately oxidized GO can directly act as a photocatalyst for various redox reactions [42].

**Figure 1:**
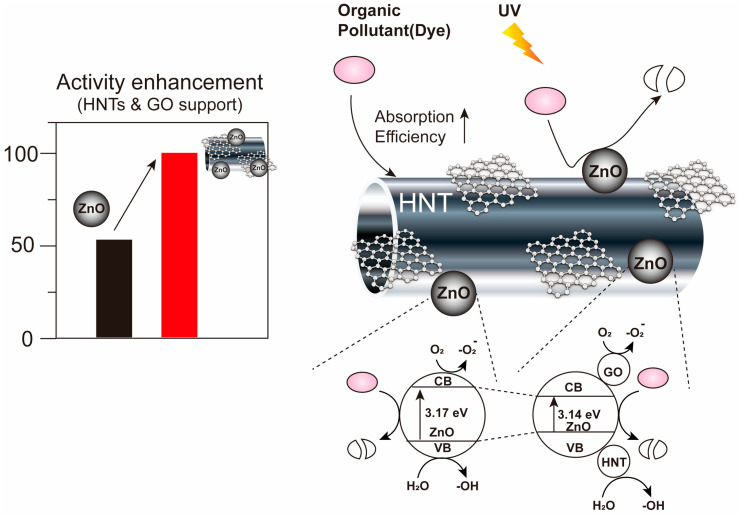
Design of ZnO/GO@HNTs and their activity enhancement. HNTs and GO can improve the absorption efficiency between the organic pollutant and the photocatalysts.

In this study, we present a novel approach to enhancing the photocatalytic activity of ZnO/HNT composites by fabricating a ZnO/GO nanocomposite on HNTs (ZnO/GO@HNT) (Figure 1). The incorporation of graphene oxide (GO) in the composite aims to improve the adsorption efficiency of dyes by leveraging the unique properties of GO, including its hexagonal carbon structure with sp2 bonds, high surface area, excellent electron mobility, and remarkable absorption stability [43]. First, we synthesized ZnO/GO@HNT nanocomposites using a sonochemical method. We analyzed the nanocomposites’ structure using XRD, Raman spectroscopy, FT-IR, and EDX-TEM. To evaluate the photocatalytic performance, we selected rhodamine b (RB) as the model organic dye, as its degradation can be easily monitored through fluorescence [44,45,46]. Under UV light irradiation, we compared the photocatalytic activity of RB when mixed with ZnO, ZnO@HNTs, ZnO/GO, and ZnO/GO@HNTs. The results revealed that while ZnO nanoparticles alone achieved a fluorescence reduction of only 56% within one hour, our ZnO/GO@HNT nanocomposites exhibited an impressive 95% reduction in the same time period, demonstrating significantly enhanced photocatalytic efficiency. Furthermore, the ZnO/GO@HNT nanocomposites displayed exceptional recyclability, as they could be reused multiple times without experiencing a significant loss in catalytic efficiency. This property further highlights the potential of our nanocomposites for practical applications, as their stable and efficient performance can contribute to the development of sustainable photocatalytic systems for the degradation of organic dyes in wastewater treatment. The successful fabrication of ZnO/GO@HNT nanocomposites offers a promising avenue for improving the photocatalytic activity of ZnO/HNT composites, opening up new possibilities for addressing water contamination issues caused by organic dyes and advancing the development of eco-friendly wastewater treatment processes.

## 2. Materials and Methods

### 2.1. Chemicals and Materials

Halloysite nanotubes (HNTs) were purchased from Sigma Aldrich (30–70 nm × 1–3 μm, nanotube, Cat. #: 1332-58-7). Zinc nitrate hexahydrate (96%, Cat. #: 37340-1201) and RB (Cat. #: 87180-1510, Chemical pure grade) were purchased from Junsei Chemical Co., Ltd. (Tokyo, Japan). Isopropyl alcohol (Cat. #: 5035-4404, 99.5%) was purchased from Daejung Chemicals & Metals Co., Ltd. (Siheung-si, Republic of Korea). Nitric acid (Cat. #: 005N0517, 54.0–55.5%) was purchased from Samchun Pure Chemical Co., Ltd. (Pyeongtaek, Republic of Korea). Ethanol (Cat. #: 220710990, 95%) was purchased from OCI. Graphene oxide (powder, 4–10% edge-oxidized) was purchased from Sigmaaldrich (St. Louis, MI, USA).

### 2.2. Purification of HNTs via Supernatant Method

To prepare the HNTs, 1 mg of HNTs was added to 50 mL of deionized (DI) water under continuous stirring for a duration of 2 h. During this stirring period, the HNTs dispersed in the water. Next, the HNT solution was combined with (NaPO_3_)6 and 10 wt % of NaOH solution, adjusting the pH to 9, and stirred for an additional 24 h. Following the stirring process, the mixture was left to rest undisturbed for 6 h.

To separate the HNTs from the solution, the sample underwent centrifugation at 3000 rpm for 5 min. As a result, the majority of the HNTs settled in the supernatant, while the remaining solution was discarded. Subsequently, the obtained precipitates were subjected to further centrifugation at 7000 rpm for 10 min. This step helped to consolidate the separation of the HNTs. After centrifugation, the precipitates were washed repeatedly with DI water through three cycles of centrifugation.

To ensure the removal of any residual impurities, the washed precipitates were dried at 60 °C overnight under vacuum conditions. This drying process eliminated any remaining moisture, resulting in the production of dry HNTs ready for further use in subsequent experiments or applications.

### 2.3. Prepearation of ZnO via Co-Precipitation Method

To synthesize the ZnO nanoparticles, 0.88 g of Zn(NO_3_)_2_ and 5.5 g of NaOH were added to 200 mL of ethanol (EtOH) and stirred for 15 min. The purpose of this step was to facilitate the dissolution and mixing of Zn(NO_3_)_2_ and NaOH in the solvent.

The resulting mixed solution was then heated on a hot plate at 60 °C for a period of 8 h. This elevated temperature and prolonged heating duration allowed for the reaction between Zn(NO_3_)_2_ and NaOH, leading to the formation of ZnO nanoparticles.

After the reaction, the precipitates formed during the heating process were collected and purified through centrifugation. In the first centrifugation cycle, the precipitates were washed with EtOH to remove any residual impurities or by-products from the reaction. Subsequently, in the following two centrifugation cycles, the precipitates were washed with DI water to ensure the removal of any remaining impurities or reaction residues.

Once the purification steps were completed, the particles were dried at 80 °C overnight under vacuum conditions. This drying process served to remove any remaining solvent or moisture, resulting in the production of dry and pure ZnO nanoparticles ready for further characterization or utilization in subsequent experiments.

### 2.4. Preparation of GO via Hummer’s Method

To prepare GO, a mixture of 0.1 g of graphite powder and 0.05 g of NaNO_3_ was added to 2 mL of H_2_SO_4_. The resulting solution was stirred for 30 min in an ice bath, ensuring a controlled temperature environment. Following this, 1 mg of KMnO_4_ was introduced to the solution, and the stirring process continued for 1 h at 35 °C in a water bath. This step facilitated the oxidation of graphite, leading to the formation of graphite oxide. To terminate the reaction, 50 mL of DI water and 1 mL of H_2_O_2_ were added to the solution. This quenching process halted the chemical reactions and stabilized the mixture. Next, the solution was subjected to centrifugation to separate the solid precipitate from the liquid. The precipitate was then washed three times with 10% HCl solution to remove impurities and excess reactants. Subsequently, the precipitate underwent additional washing with DI water until the solution reached a pH of 7, ensuring the removal of any residual acid or salts. The resulting product, GO, was then dried for a duration of 3 days under freezing vacuum conditions. This prolonged drying period allowed for the complete removal of any remaining solvent or moisture, resulting in the formation of dry graphite oxide powder suitable for further use or characterization.

### 2.5. Preparation of ZnO/GO@HNT Nanocomposites via Sonochemical Synthesis

**Figure 2:**
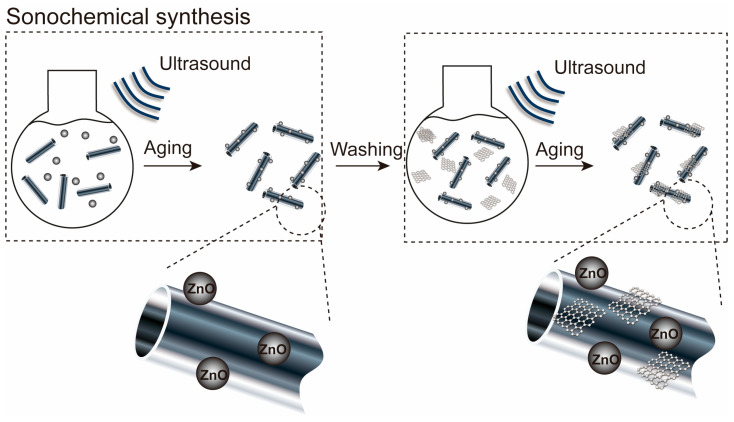
Synthesis processes of the ZnO/GO@HNT nanocomposites. The ZnO/GO@HNT nanocomposites were prepared using an ultrasound-assisted wet impregnation method.

HNTs (0.03 g) were dispersed in DI water (40 mL) to form an HNT suspension after 1 h of sonication. ZnO (0.015 g) was slowly dissolved in the HNT suspension via stirring for 30 min and sonication for 30 h. Then, the composites were centrifuged and washed several times with EtOH and DI water and dried at 80 °C for 16 h. The synthesized ZnO/HNT nanocomposites (0.02 g) were dispersed in DI water (40 mL) to dissolve the ZnO/HNT suspension via sonication for 30 min. GO (0.01 g) was successively dissolved into the nanocomposites by stirring for 1 h and sonicating for 90 min. Then, the ZnO/GO@HNT solution was centrifuged, washed several times with EtOH and DI water, and dried at 60 °C for 18 h. Figure 2 shows the synthesis processes of the ZnO/GO@HNT nanocomposites. The process of immobilizing ZnO on HNTs involves two reactions: (1) hydrolysis of the precursor in either acidic or basic conditions, and (2) polycondensation of the hydrolyzed products. The titanium precursors undergo hydrolysis, resulting in the formation of nanoparticles in the form of a sol. These nanoparticles then grow by attaching themselves to the surface of HNTs through the gelation of the sol.

### 2.6. Photocatalytic Activity of Organic Dye

The photocatalytic performance of the nanocomposites was evaluated using RB under dark exposure to UV irradiation, provided by a 250 W high-pressure UV lamp (Ushio Inc., Tokyo, Japan, UXM-Q256BY). A glass vial with a capacity of 15 mL was used as the photoreactor vessel. The nanocomposites and ZnO nanoparticles were used for the photocatalytic degradation of RB, and their efficacies were compared. These experiments used 3 mg of photocatalytic material and 7 ppm of dye solution (15 mL) and were performed at room temperature at pH 5 with UV light.

In order to assess the degradation of RB dye through photocatalysis, the initial dye concentration was varied, and 3 mg of ZnO/GO@HNTs was added to each solution (15 mL) at a concentration of 7 ppm. The mixture was stirred in a dark environment for 30 min to ensure adequate adsorption. Following adsorption, the sample was subjected to UV irradiation using a 250 W, 365 nm UV lamp (Ushio-SP9) while maintaining continuous stirring. A UV–Vis spectrometer was employed to measure the absorbance of the dye as it decomposed over time. The degradation efficiency of the dye was determined using the equation [47,48]

(1) $$\mathrm{Degradation}\;\mathrm{efficiency}\;(\%)=\frac{C_{0}-C}{C_{0}}\;\times \;100$$

where C_0_ represents the initial concentration of RB (mg/L), and C denotes the decomposed concentration of the dye. Additionally, the rate constant (k_1_) was derived using the pseudo-first-order kinetic model:

(2) $$ln\frac{C_{0}}{C}=k_{1}t$$

For comparative purposes, 7 ppm of catalysts, including ZnO, ZnO/HNT, ZnO/GO, and ZnO/GO@HNT composites, was added to a 15 mL solution of RB, followed by 30 min of stirring in darkness. Subsequently, the samples were exposed to UV irradiation, and the changes in absorbance during the dye decomposition process were recorded. The removal efficiencies and decomposition rate constants for each catalyst were calculated.

### 2.7. Measurement

Scanning electron microscopy (FE-SEM, hitachi, s-4800) was performed, which uses a type of electron microscope that produces images of a sample by scanning the surface with a focused beam of electrons. The electrons interact with atoms in the sample, producing various signals that contain information about the sample’s surface morphology and composition. Energy-dispersive X-ray spectroscopy analysis was also performed in conjunction with SEM. Fourier-transform infrared spectroscopy (FTIR, BK Instruments Inc., Delhi, India, FTLA2000) was used, which measures the infrared spectrum of absorption, emission, photoconductivity, or Raman (Kaiser Optical Systems Inc., Ann Arbor, MI, USA, Hololab Series 5000) scattering of a solid, liquid, or gas. The FTIR spectrometer simultaneously collected spectral data in a wide range, measuring the intensity over a narrow range of wavelengths at a specific time. FTIR has made dispersive infrared spectrometers all but obsolete, opening up new applications of infrared spectroscopy. High-resolution X-ray diffraction (XRD, Bruker, D-8 Discover) analysis was performed using a diffractometer with copper source radiation, running at 40 kV and 40 mA and scanning from 4° to 80° at 0.02°. UV–Vis spectra for the supernatant of the RB solution were obtained using a UV–visible spectrophotometer (Evolution 60S, Thermo Fisher Scientific, Waltham, MA, USA).

## 3. Results and Discussion

### 3.1. Morphology of Nanocomposites

**Figure 3:**
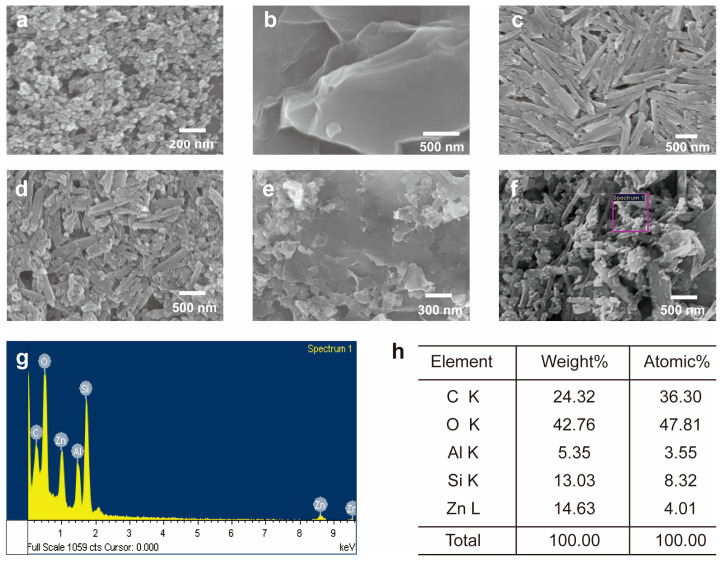
SEM images of (**a**) ZnO nanoparticle, (**b**) GO, (**c**) HNTs, (**d**), ZnO/HNT, (**e**) ZnO/GO, and (**f**) ZnO/GO@HNTs. (**g**) EDS mapping spectra of the area indicated by the box in (**f**). (**h**) Relative atomic amounts from EDS.

To observe the size and shape of the nanomaterials (ZnO, GO, and HNTs) and to verify the formation of the composites (ZnO/HNT, ZnO/GO, and ZnO/GO@HNT), we characterized the materials and composites using scanning electron microscopy (SEM). The SEM images of ZnO (Figure 3a) showed aggregated ZnO particles with a ~20 nm diameter. The layered sheet shape of GO and the pipe-like structure of the HNTs were also observed (Figure 3b,c). The SEM images of ZnO/HNT and ZnO/GO@HNT confirmed that the ZnO nanoparticles were attached to the HNTs (Figure 3d,e). Bright spots on the composites and sheet-type aggregates were observed in the ZnO/GO@HNT images, presumably corresponding to GO. SEM-EDS analysis underpinned the existence of Si, Zn, Al, O, and C atoms on the surface of ZnO/GO@HNT (Figure 3g,h).

### 3.2. Crystal Structure and Functional Groups of the Nanocomposites

**Figure 4:**
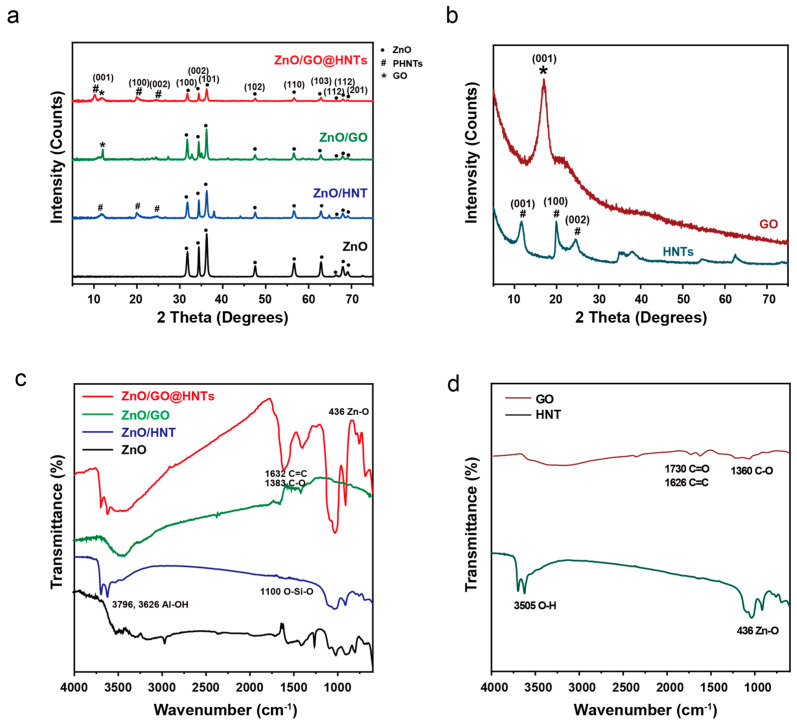
(**a**) XRD patterns and (**c**) FT-IR spectra of prepared ZnO/GO@HNTs, ZnO/GO, and ZnO/HNTs. (**b**) XRD patterns and (**d**) FT-IR spectra of GO and HNTs.

**Figure 5:**
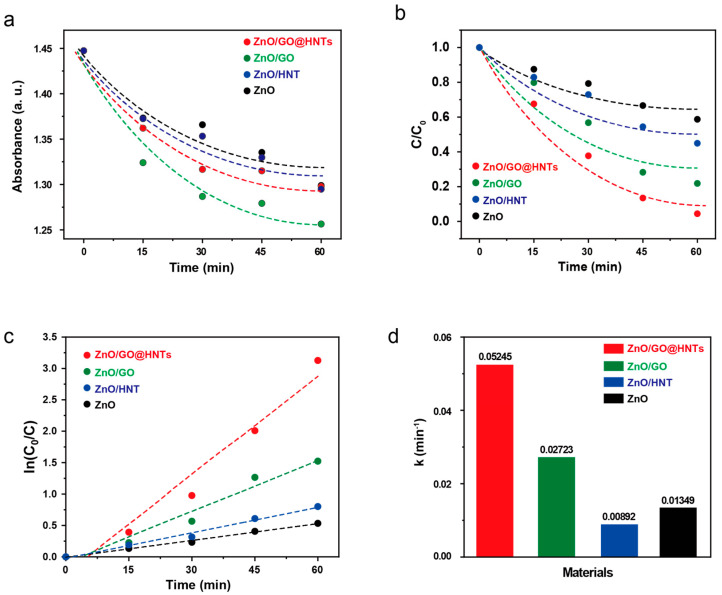
(**a**) Absorption and (**b**) photodegradation of RB by HNTs, ZnO, ZnO/GO, and ZnO/GO@HNTs as a function of time. (**c**) Pseudo-first-order kinetic curves of the nanocomposites and (**d**) their kinetic constants.

To investigate the microstructures of ZnO, GO, HNTs, and the nanocomposites, we performed XRD. The XRD pattern of the ZnO nanoparticles showed nine peaks, which can be indexed to the (100), (002), (101), (102), (110), (103), (200), (112), and (201) planes (JCPDS card number = 36–1451). The fabricated ZnO nanoparticles showed the typical hexagonal structure (lattice constants of a = b = 3.25 Å, c = 5.20 Å), indicating the formation of nanoparticles. All the peaks in the spectra of the ZnO/GO@HNT nanocomposites could be indexed to the (001), (100), (002), (101), (102), (110), (103), (200), (112), and (201) planes, showing an overlap of the typical GO (001) and HNT peaks (11.6° (001), 19.9° (100), and 24.9° (002)). These overlapped peaks indicate that the ZnO nanoparticles and GO were attached to the ZnO/GO@HNTs. Based on the XRD results, the hexagonal structure of the ZnO nanoparticles in the ZnO/GO@HNTs had a lattice structure (a = b = 5.09 Å, c = 7.48 Å). We assume that the increase in the d-spacing of ZnO on the ZnO/GO@HNTs compared to the isolated ZnO nanoparticles originated from the intercalation by GO and the HNTs (Figure 4a,b). The functional groups present in the various materials, including the HNTs, HNTs/ZnO, GO/HNTs/ZnO, GO, ZnO, and GO/ZnO, were analyzed using FTIR spectroscopy at room temperature (Figure 4c,d). The first observed peak at 436 cm^−1^ corresponds to the characteristic vibration mode of ZnO (Figure 4a). The second set of peaks at 1360 cm^−1^, 1626 cm^−1^, and 1730 cm^−1^ are attributed to C-O, C=C, and C=O vibrations, respectively, which indicate the presence of carboxylate anions absorbed on the surface of GO (Figure 5b). Additionally, the broad absorption peak at 3505 cm^−1^ indicates the presence of OH groups. In the case of the HNTs (Figure 5a), the absorption bands at 3796 cm^−1^ and 3626 cm^−1^ are assigned to the O-H stretching of the inner-surface hydroxyl groups (Al-O-H), while the band at 914 cm^−1^ is attributed to the O-H deformation of hydroxyl groups. Moreover, the absorption bands at 1083 cm^−1^ and 1025 cm^−1^ correspond to in-plane stretching vibrations of O-Si-O, and the band at 1100 cm^−1^ represents O-Si-O perpendicular stretching. The bending vibration of Si-O and the stretching vibration of Al-O can be observed at 536 cm^−1^ and 462 cm^−1^, respectively. The observed bands in the ZnO/GO@HNTs are attributed to the different vibrational modes of Al-OH, C=C, Si-O, and Zn-O, with peaks observed at 3696 cm^−1^, 1627 cm^−1^, 1100 cm^−1^, and 436 cm^−1^, respectively, confirming the formation of a nanocomposite (Figure 4c).

We also performed Raman analysis (Figure S1) to understand the structure of GO on our nanocomposites. GO exhibits two characteristic bands: the D band at 1354 cm^−1^, corresponding to the breathing mode of six-membered carbon rings, and the G band at 1595 cm^−1^, corresponding to the in-plane vibration of sp^2^ carbon pairs. In the micro-Raman spectra of GO, HNTs, ZnO, ZnO//GO, ZnO/HNT, and ZnO/GO@HNTs, the spectra of the HNTs showed Al-O (257 cm^−1^) and Si-O peaks (487 cm^−1^), and those of ZnO exhibited Zn-O peaks at 343 and 437 cm^−1^ (Figure S1a). In the Raman analysis, the D band, G band, and Al, Si, and Zn peaks confirmed the formation of the nanocomposite (Figure S1b).

### 3.3. Absorption and Photocatalytic Degradation of RB

High-photocatalytic degradation experiments were carried out using our composite materials. The RB molecules could bind to ZnO, ZnO/GO, ZnO/HNTs, and ZnO/GO@HNTs in dark conditions without photodegradation. We tracked the absorbance change in the mixed solution of RB and the nanocomposites to measure the amount of RB on the nanocomposites (Figure 5a). Among the nanocomposites tested, ZnO/GO collected the most RB molecules, showing a sharp decrease in absorbance. This high absorption efficiency is due to ZnO/GO possessing the highest content of GO; the RB molecules can bind to GO through π–π interactions. However, the ZnO/GO@HNTs showed the highest photocatalytic efficacy under UV light irradiation, although they did not exhibit the highest absorption efficiency (Figure 5b). Although the ZnO/HNTs effectively collected RB molecules, the aggregation of the ZnO/HNTs hindered effective photocatalysis. However, since the HNTs acted as supporting materials to prevent the aggregation of the nanocomposites in the ZnO/GO@HNTs, they achieved the highest photocatalytic efficiency. Photocatalysis using the ZnO/GO@HNTs achieved 95% degradation of RB molecules in 1 h under UV radiation (Figure 5b), whereas the ZnO nanoparticles degraded only 56% of the dye under the same conditions. On the other hand, various nanomaterials as a photocatalyst have been often used for degradation of rhodamine B. Their photocatalytic activities were summarized and compared with our results in Table S1. Various In addition, the calculated kinetic constant of the ZnO/GO@HNTs, obtained from the kinetic velocity equation (Figure 5c), was 0.05245 min^−1^ (Figure 5d). During the photocatalysis of ZnO/GO@HNTs, the electrons of ZnO are supported by the abundant electrons of GO, and the holes of ZnO are supported by the negative charge on the surface of the HNTs [49,50].

**Figure 6:**
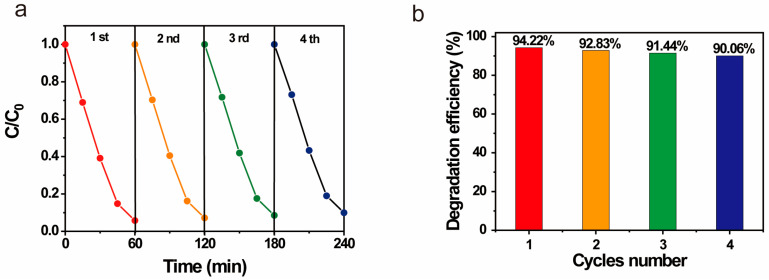
Recycling of ZnO/GO@HNTs for photocatalytic degradation of RB: (**a**) concentration rate and (**b**) degradation efficiency.

Additionally, we performed recycling experiments on our photocatalyst. After each photodegradation process under UV light, the ZnO/GO@HNT nanocomposites were separated via centrifugation, dried, and reused. The photolysis efficiency was maintained at over 90% over four reuse cycles, demonstrating the reusability of this nanocomposite using a simple separation method (Figure 6).

### 3.4. Band Gap of Photocatalysis and Mechanism

**Figure 7:**
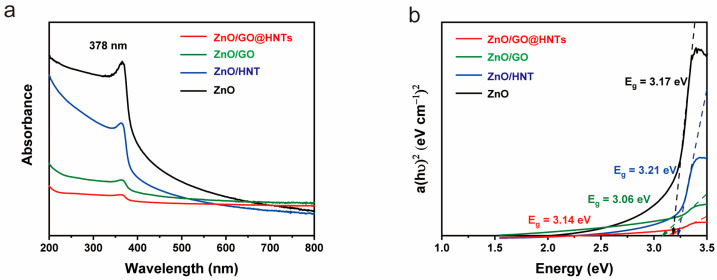
(**a**) UV–visible spectra and (**b**) band gaps from the Tauc plots of ZnO, ZnO/HNTs, and ZnO/GO.

UV–visible spectra were measured for ZnO, ZnO/HNTs, ZnO/GO, and ZnO/GO@HNTs, and the band gaps were determined using the Tauc plot equation. The calculated band gap values were 3.17 eV, 3.24 eV, 3.11 eV, and 3.14 eV for ZnO, ZnO/HNTs, ZnO/GO, and ZnO/GO@HNTs, respectively (Figure 7). These results indicate that the materials’ electrical properties for photocatalysis are within the semiconductor range, suggesting their potential for photocatalysis. During photocatalysis activity, the holes of ZnO are effectively supported by the negative charge on the surface of the HNTs, while the electrical properties of GO facilitate the transfer of electrons. This enhanced electron transfer rate leads to a reduction in the band gap of the nanocomposite.

The proposed photocatalytic process can be described as follows:

(1) GO/HNTs/ZnO + *hv* → GO (e^−^) + HNTs (h^+^)
(2) GO (e^−^) + O_2_ → **^·^**O_2_^−^
(3) HNTs (h^+^) + H_2_O → OH· + H^+^
(4) **^·^**O_2_^−^ + H_2_O → HO_2_· + OH^−^
(5) HO_2_· + H_2_O → H_2_O_2_ + OH·
(6) H_2_O_2_ + *hv* → 2OH·
(7) Dye + h^+^ + **^·^**O_2_^−^ + OH· → CO_2_ + H_2_O

When exposed to light, ZnO//GO@HNTs separate into electrons and holes. The electrons react with oxygen from the organic dyes, forming radical oxygen. This radical oxygen then reacts with water, generating hydroxyl radicals. The primary pathway involves the hydroxyl radicals reacting solely with hydroxyl groups, while in another pathway, hydroxyl radicals interact with the dye molecules, resulting in their degradation into carbon dioxide and water.

## 4. Conclusions

In this study, we successfully synthesized nanocomposites of ZnO/GO@HNTs using a sonochemical method. The synthesized materials were characterized using various techniques, including XRD, SEM, EDX, UV, PL, FT-IR, and Raman spectroscopy. By taking advantage of the electrical properties of GO and the aggregation-preventing properties of HNTs as supports, our ZnO-based photocatalysts exhibited enhanced photocatalytic activity. To evaluate the effectiveness of our nanocomposites, photocatalytic experiments were conducted under UV irradiation for a duration of 60 min. The results showed that the nanocomposites achieved a remarkable 95% photodegradation of RB (a representative dye), while the ZnO nanoparticles alone achieved only 56% dye degradation under the same experimental conditions. Through the electrical properties of GO and the aggregation prevention of the HNT supports, our ZnO-based photocatalysts can provide enhanced photocatalytic activity. Furthermore, the nanocomposites demonstrated excellent photocatalytic activity and maintained a stable performance throughout four recycling experiments. This suggests their potential for long-term and efficient use in the purification of aquatic environments contaminated with organic pollutants. Consequently, our ZnO/GO@HNT nanocomposites offer an environmentally friendly solution for the textile industry and its wastewater treatment, contributing to a more sustainable approach to addressing the challenges of organic pollutant removal.

## Data Availability

The data presented in this study are available in this article and the Supplementary Materials.

## Supplementary Materials

The following supporting information can be downloaded at: https://www.mdpi.com/article/10.3390/nano13131895/s1, Figure S1: Rman spectrum of nanocomposites of (a) HNT, ZnO, and ZnO/HNT, and (b) ZnO/GO and ZnO/GO/HNTs. Table S1: Summary of the photocatalytic rhodamine B degradation of the various reported materials at direct sunlight. References [51,52,53,54,55,56,57] are cited in the Supplementary Materials.
