# Supplementary material for: ZnO/Graphene Oxide on Halloysite Nanotubes as a Superabsorbent Nanocomposite Photocatalyst for the Degradation of Organic Dyes
Source: Nanomaterials (Basel). 2023 Jun 21;13(13):1895. doi: 10.3390/nano13131895 (PMC10343214; doi:10.3390/nano13131895)
Supplement: Supplementary file 1 [file nanomaterials-13-01895-s001.zip › nanomaterials-2460777-supplementary.pdf]

Supplementary Materials

ZnO/Graphene Oxide on Halloysite Nanotubes as a Superabsorbent Nanocomposite Photocatalyst for the Degradation of Organic Dyes

Jongik Park [1], Hyungwook Lee [2], Keonku Lee [2], Sieun Noh [2], Soyeong Jin [2,3], Jungho Jae [4], Youngdo Jeong [3,5,*] and Jaegeun Noh [1,2,6,*]

[1] Department of Convergence of Nanoscience, Hanyang University, 222 Wangsimni-ro, Seongdong-gu, Seoul 04763, Republic of Korea; siy171@naver.com

[2] Department of Chemistry, Hanyang University, 222 Wangsimni-ro, Seongdong-gu, Seoul 04763, Republic of Korea; winning_07@naver.com (H.L.); leekku10@gmail.com (K.L.); sieun.noh@kakao.com (S.N.); truejin@hanyang.ac.kr (S.J.)

[3] Center for Biomaterials, Biomedical Research Institute, Korea Institute of Science and Technology (KIST), Seoul 02792, Republic of Korea

[4] School of Chemical and Biomolecular Engineering, Pusan National University, Busan 46241, Republic of Korea; jh.jae@pusan.ac.kr

[5] Department of HY-KIST Bio-Convergence, Hanyang University, 222 Wangsimni-ro, Seongdong-gu, Seoul 04763, Republic of Korea

[6] Research Institute for Convergence of Basic Science, Hanyang University, 222 Wangsimni-ro, Seongdong-gu, Seoul 04763, Republic of Korea

* Correspondence: zerodegree@kist.re.kr (Y.J.); jgnoh@hanyang.ac.kr (J.N.)

Instruments

TEM samples were prepared by placing one drop of the desired catalysts solution (~1–3 μM) onto a 300-mesh copper grid coated with carbon film, followed by drying. The samples were analyzed and photographed using a JEM 2100F (JEOL) transmission electron microscope at an accelerating voltage of 80 kV.

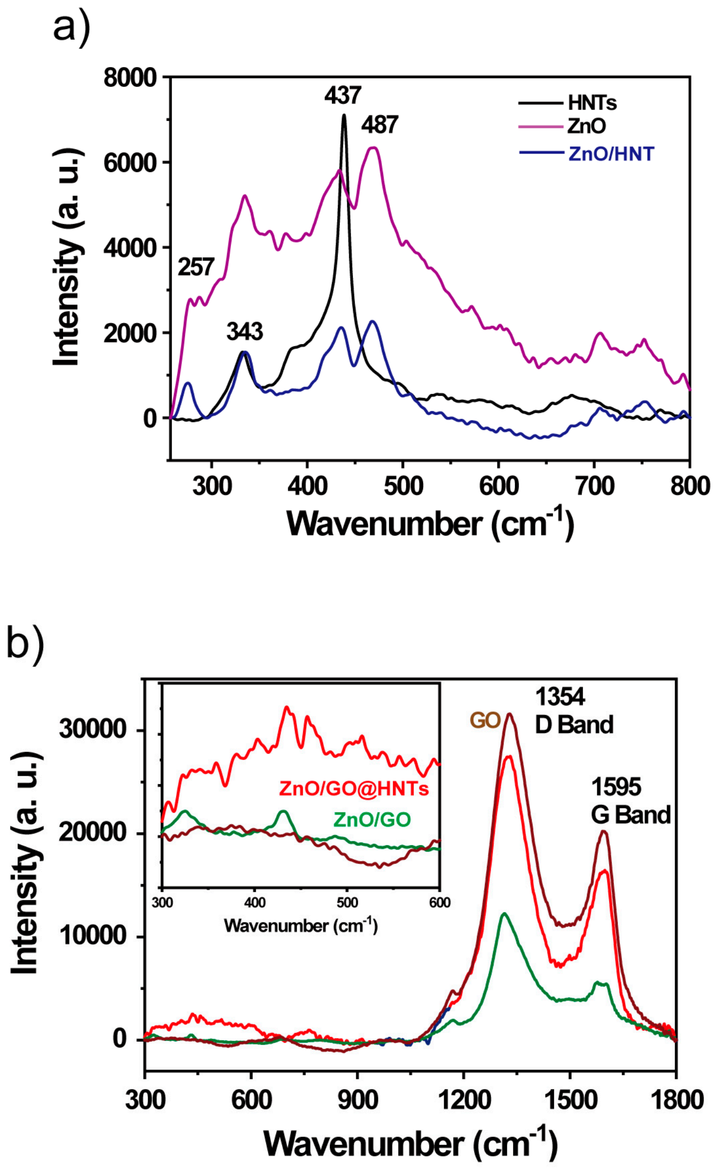

Figure S1. Raman spectrum of the nanocomposites of (a) HNT, ZnO, and ZnO/HNT, and (b) ZnO/GO and ZnO/GO/HNTs.

Table S1. Summary of the photocatalytic rhodamine B degradation of the various reported materials at direct sunlight.

Entry	Materials	Concentration	Dosing	Time	Degradation %	Ref.
1	Mn, Fe, Cu, Ce, Ag, Zr doped TiO_2	10 mg/L 80 mL	20 mg	60 min	Mn/TiO_2 94.3% Fe/$TiO_2$90.9% Cu/TiO_2 82.5% Ce/TiO_2 77.1% Ag/TiO_2 74.9% Zr/TiO_2 69.7%	[51]
2	Ag, Co, Ni, Pd doped CCA/TiO_2	10 mg/L 100 mL	100 mg	180 min	Pd/CCA/TiO_2 97.5% Ag/CCA/TiO_2 95.5% Co/CCA/TiO_2 81.8% Ni/CCA/TiO_2 78.8%	[52]
3	TiO_2/g-C_3N_4	10 mg/L 30 mL	20 mg	40 min	91.26%	[53]
4	TiO_2/α-Fe_2O_3	1.0×10^{-5} M (5 mg/L) 5 mL	10 mg	60 min	98.0%	[54]
5	$LaFeO_3$/Ag_2CO_3	1.0×10^{-5} M (5 mg/L) 80 mL	80 mg	45 min	99.5%	[55]
6	g-C_3N_4/TiO_2/Bi_2O_3	20 mg/L 50 mL	50 mg	120min	colorless	[56]
7	TiO_2/Polyfluorene	6.0×10^{-5} M 75 mL	10 mg	60 min	colorless	[57]
8	HNTs/TiO_2/La	5 mg/L 20 mL	20 mg	120 min	96.6%	[47]
9	ZnO/GO@HNT	7 ppm	3 mg	60 min	95%	This work

[51] Feng, H., Zhang, M., Yu, L. E., 2012. Hydrothermal synthesis and photocatalytic performance of metal-ions doped TiO_2. Appl. Catal. A Gen. 413–414, 238–244.

[52] Mahlambi, M. M., Mishra, A. K., Mishra, S. B., Krause, R. W., Mamba, B. B., Raichur, A. M., 2013. Effect of metal ions (Ag, Co, Ni, and Pd) on the visible light degradation of rhodamine B by carbon-covered alumina supported TiO_2 in aqueous solutions. Ind. Eng. Chem. Res. 52, 1783–1794.

[53] Adhikari, S. P., Awasthi, G. P., Kim, H. J., Park, C. H., Kim, C. S., 2016. Electrospinning directly synthesized porous TiO_2 nanofibers modified by graphitic carbon nitride sheets for enhanced photocatalytic degradation activity under solar light irradiation. Langmuir 32, 6163−6175.

[54] Mahajan. J., Jeevanandam, P., 2018. Synthesis of TiO_2@a-Fe_2O_3 core–shell heteronanostructures by thermal decomposition approach and their application towards sunlight driven photodegradation of rhodamine B, New J. Chem. 42, 2616.

[55] Pirzada, B. M., Pushpendra, Kunchala, R. K., Naidu, B. S., 2019. Synthesis of $LaFeO_3$/Ag_2CO_3 nanocomposites for photocatalytic degradation of rhodamine B and p-Chlorophenol under natural sunlight. ACS Omega 4, 2618–2629.

[56] Peng, W., Yang, C., Yu, J., 2020. Bi_2O_3 and g-C_3N_4 quantum dot modified anatase TiO_2 heterojunction system for degradation of dyes under sunlight irradiation. RSC Adv. 10, 1181–1190.

[57] Bai, W., Tian, X., Yao, R., Chen, Y., Lin, H., Zheng, J., Xu, Y., Lin, J., 2020. Preparation of nano-TiO_2@polyfluorene composite particles for the photocatalytic degradation of organic pollutants under sunlight. Sol. Energy 196, 616–624.
